# Curcumin: A Magical Small Molecule with a Large Role in Active-Intelligent Degradable Food Packaging

**DOI:** 10.3390/ijms26083917

**Published:** 2025-04-21

**Authors:** Di Wang, Siyu Zhou, Nan Li, Dehui Lin

**Affiliations:** Shaanxi Engineering Laboratory for Food Green Processing and Safety Control, Shaanxi Key Laboratory for Hazard Factors Assessment in Processing and Storage of Agricultural Products, College of Food Engineering and Nutritional Science, Shaanxi Normal University, Xi’an 710062, China

**Keywords:** curcumin, active-intelligent food packaging, antibacterial activity, antioxidant activity, pH indicator

## Abstract

Curcumin exhibits antioxidant, antibacterial, antitumor, and anti-inflammatory biological properties. Its dual functionality as both a food additive and a pH-sensitive colorant has led to extensive applications in meat products and other food systems, thereby garnering significant research interest. In recent years, curcumin-loaded active-intelligent food packaging films have emerged as a promising innovation due to their multifunctional capabilities: not only do they prevent microbial contamination and extend food shelf life, but they also enable real-time freshness monitoring through visual colorimetric responses. This paper first delineates the molecular structure and fundamental biological mechanisms of curcumin. Subsequently, it systematically reviews the strategies for curcumin incorporation (including encapsulation techniques and composite formulations) and advanced fabrication methodologies for developing active-intelligent biodegradable films. Finally, the current applications of curcumin in polymer-based smart packaging systems are critically analyzed, with prospective research directions proposed to address existing technological limitations.

## 1. Introduction

Food packaging, a kind of wrapping on the surface of food, is used for the containment and preservation of food quality, as well as the protection of food from environmental, physical, and microbiological factors [1]. Conventional petroleum-derived plastic packaging materials (e.g., polyethylene terephthalate, polypropylene, polystyrene) exhibit significant limitations, such as non-biodegradability, leaching of toxic compounds (e.g., bisphenol A and phthalates), and microplastic generation [2,3,4,5], which brings great pressure for environmental governance and causes certain health hazards to the human body, thus it cannot completely satisfy the growing demands of consumers for high quality foods and environmental protection [2,6]. Consequently, biodegradable packaging materials derived from biopolymers (e.g., polysaccharides, proteins, lipids) and their composites have garnered considerable research interest [7]. However, the challenge of food spoilage cannot be addressed solely through biodegradable packaging solutions [8]. Bioactive ingredients, such as antibacterial agents, antioxidants, deoxidizers, hygroscopic agents, ethylene scavengers and carbon dioxide emitters, are selectively added to biodegradable base materials to prepare active packaging, which can prolong the shelf life of food [9]. With growing food safety awareness and evolving consumer preferences, modern packaging must not only extend shelf-life but also provide real-time quality indication [4,10,11]. Consequently, responsive elements, including time-temperature indicators, gas sensors, and pH-sensitive dyes, have been integrated into biodegradable substrates to create intelligent packaging systems [12]. These systems dynamically interact with the internal environment to monitor critical parameters (e.g., storage duration, temperature history, and freshness status), thereby enhancing food safety assurance [10,13,14].

Despite these advances, conventional biodegradable packaging with limited functionality remains inadequate to meet contemporary consumer expectations regarding food quality and safety [15]. In contrast, multifunctional packaging systems integrating advanced technologies enable more comprehensive monitoring of food storage conditions, demonstrating significant potential for commercial applications [16]. Consequently, active-intelligent packaging systems have gained substantial research interest due to their dual capacity to preserve food quality while providing real-time safety indicators [9]. The fabrication of active-intelligent packaging requires the incorporation of bioactive natural compounds, including essential oils (e.g., thyme oil), polyphenols (e.g., curcumin, quercetin), and botanical extracts (e.g., anthocyanins) [17,18,19,20,21,22]. Notably, the inherent instability of many bioactive compounds often compromises the responsiveness of functionalized packaging films [23], presenting significant challenges in developing highly sensitive active-intelligent systems.

Curcumin, a natural polyphenolic compound, exhibits dual functionality as a potent antioxidant and demonstrated antimicrobial agent [24]. Notably, curcumin displays pH-responsive chromic behavior, undergoing distinct color transitions across physiological pH ranges [25,26]. Consequently, curcumin enables both food preservation and real-time quality monitoring through its multifunctional characteristics [14,27,28,29]. This multifunctionality renders curcumin particularly valuable for advanced active-intelligent packaging systems. Despite numerous investigations into curcumin-incorporated active-intelligent packaging, a systematic review elucidating its multifunctional roles remains lacking [4,14,30,31]. This review systematically examines (a) the molecular structure and physicochemical properties of curcumin, (b) its multifunctionality in biodegradable active-intelligent biodegradable packaging, (c) curcumin-loading methods, preparation methods and the corresponding effects on the properties of active-intelligent biodegradable food packaging, (d) the application of curcumin-loaded active-intelligent food packaging, (e) the possibility of curcumin release into packaged food products, the stability of packaging over time and its biodegradability, (f) an overview of the trends/challenges of active-intelligent biodegradable food packaging loaded with curcumin.

## 2. Structure, Physicochemical Properties and Biological Activities of Curcumin

**Figure 1:**
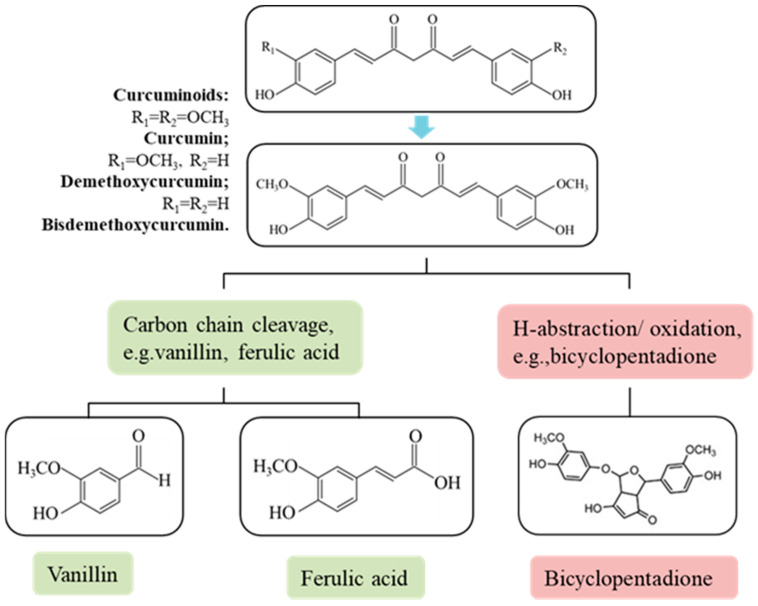
Structure and degradation of curcumin at physiological pH values in aqueous solutions [43,44].

Turmeric, derived from *Curcuma long* L. (a flowering plant of the ginger family), typically contains 2–9% curcuminoids by dry weight [32,33,34]. As the principal bioactive polyphenol, the chemical notation of curcumin is 1,7-bis(3,4-dimethoxyphenyl)-1,6-heptadiene-3,5-dione or diferuloylmethane and its chemical formula is C_21_H_20_O_6_ [35]. Curcumin in most commercial samples belongs to the curcuminoids, consisting of curcumin (77%), dimethoxy-curcumin (DMC, 17%) and dimethoxy-curcumin (BDMC, 3%) (Figure 1) [34,36,37]. Commercial curcumin preparations typically comprise three major curcuminoids: curcumin I (77%), dimethoxy-curcumin (17%), and bisdemethoxy-curcumin (3%), as illustrated in Figure 1 [38]. Curcumin exhibits marked chemical instability, particularly in aqueous media at physiological pH (t_1/2_ = 4–8 min), rapidly decomposing into multiple degradation products (Figure 1). The degradation products of curcumin include (a) alkaline hydrolysates formed by the breaking of the curcumin carbon chain, such as ferulic acid, vanillin and ferulic methane [39,40], (b) cyclization products (predominantly bicyclopentadione derivatives) formed through oxidative coupling [38,41]. Recent studies indicate that alkaline hydrolysis constitutes a minor degradation pathway, with autoxidation (primarily forming bicyclopentadione derivatives) representing the predominant decomposition route [38,42].

**Figure 2:**
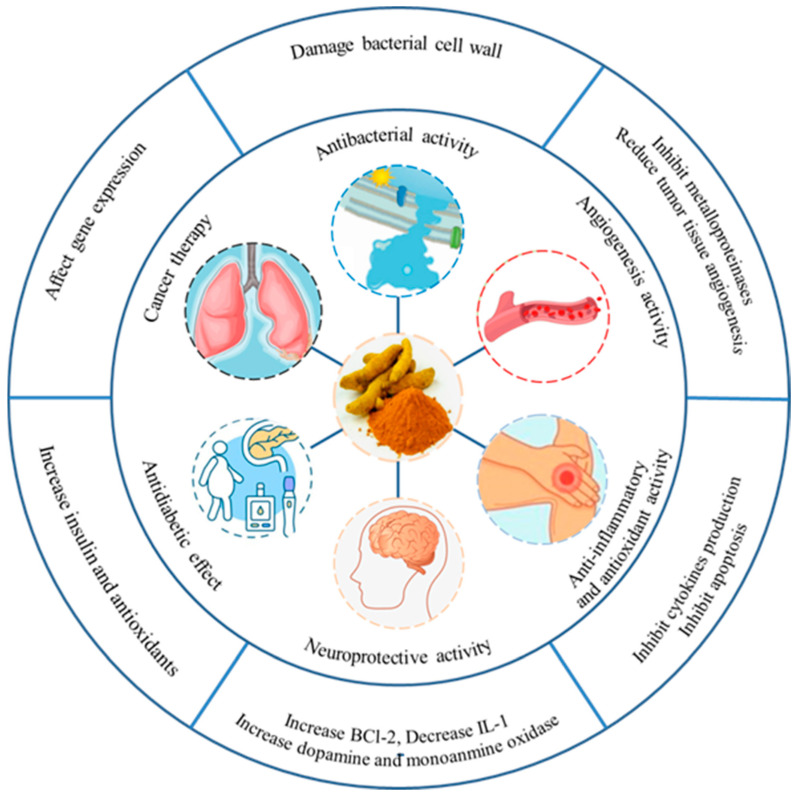
Biological properties of curcumin [50,52,53].

According to the regulations of the USA Food and Drug Administration (FDA), curcumin is generally recognized as safe (GRAS). Consequently, curcumin demonstrates excellent safety profiles even at elevated concentrations, with established no-observed-adverse-effect levels (NOAEL) [45,46,47]. Curcumin has a variety of biological properties, including antioxidant, antibacterial, anticancer, anti-tumor, anti-lipid-lowering and neuroprotective effects [48,49]. The biological properties of curcumin are shown in Figure 2 [50]. Owing to its diverse pharmacological activities, curcumin finds extensive applications across multiple industries, including pharmaceutical formulations, nutraceutical products, cosmetic preparations, and functional food development [51].

**Figure 3:**
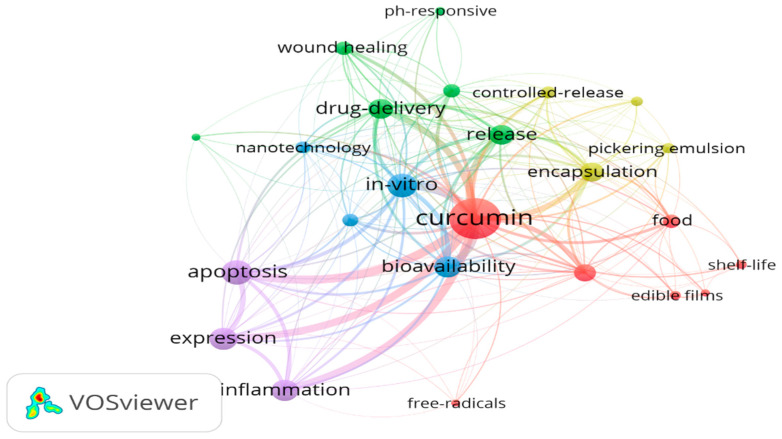
The development trend of curcumin in recent years (2019–2024). The data are based on the results from the Web of Science database by using the key search word “curcumin”, and then the network map of food packaging is plotted by VOS viewer software (version 1.6.20).

The development trend of curcumin in recent years is described in Figure 3. Here, the central node, “curcumin”, is connected to a wide range of keywords, indicating its multifaceted applications in various fields, including drug delivery, food science, and medical research. The density of connections suggests that curcumin is a highly versatile compound with significant interest across multiple disciplines. Keywords such as “drug-delivery”, “nanotechnology”, “controlled-release”, and “in-vitro” form a prominent cluster. This highlights the growing interest in curcumin as a therapeutic agent, particularly in the context of nanotechnology-based drug delivery systems. Research in this area focuses on enhancing curcumin’s bioavailability, stability, and targeted release. Future trends may include the development of advanced nano-carriers, such as liposomes and nanoparticles, to optimize curcumin’s therapeutic potential. The cluster around “food”, “shelf-life”, and “edible films” reflects curcumin’s role in food preservation and functional food development. Studies are exploring curcumin’s antimicrobial and antioxidant properties to extend shelf life and improve food safety. Keywords like “apoptosis”, “inflammation”, “free radicals”, and “wound healing” indicate curcumin’s potential in medical applications. Research in this area emphasizes curcumin’s anti-inflammatory, antioxidant, and anticancer properties. The connection between “encapsulation”, “Pickering emulsion”, and “release” suggests advancements in curcumin encapsulation technologies. These technologies aim to enhance curcumin’s stability and controlled release, which are critical for its application in both food and pharmaceutical industries. In conclusion, the keyword map provides a comprehensive overview of curcumin’s research landscape, highlighting both current trends and future opportunities. By addressing these areas, researchers can further advance curcumin’s applications across multiple fields.

## 3. The Potential Roles of Curcumin in Active-Intelligent Biodegradable Food Packaging

**Figure 4:**
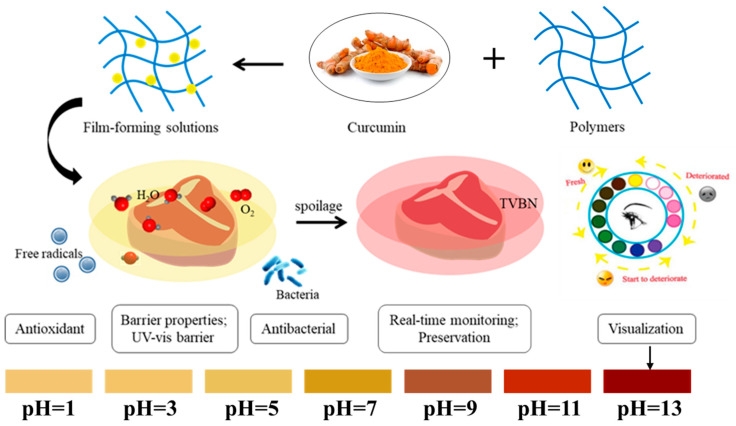
Preparation and properties of intelligent films loaded with curcumin [58,59].

Recent scientific efforts have focused on incorporating curcumin into various biodegradable polymer matrices, particularly protein- and polysaccharide-based systems (e.g., cellulose derivatives, marine biopolymers, like carrageenan and chitosan, and their nanocomposites), to engineer advanced functional films for active-intelligent packaging applications [21,31,54,55,56,57]. Figure 4 shows the main properties of curcumin-loaded films [58].

### 3.1. Acting as an Antioxidant

Food oxidation is one of the main problems of food deterioration. This process not only diminishes nutritional quality and accelerates product deterioration, but also generates potentially harmful oxidation byproducts that may pose health risks to consumers [60,61]. Consequently, significant research efforts have been directed toward developing antioxidant-incorporated packaging systems to mitigate oxidative degradation and preserve food quality [62]. Importantly, natural antioxidants are particularly attractive for developing biodegradable active packaging, as they minimize undesirable component interactions while maintaining functionality.

**Figure 5:**
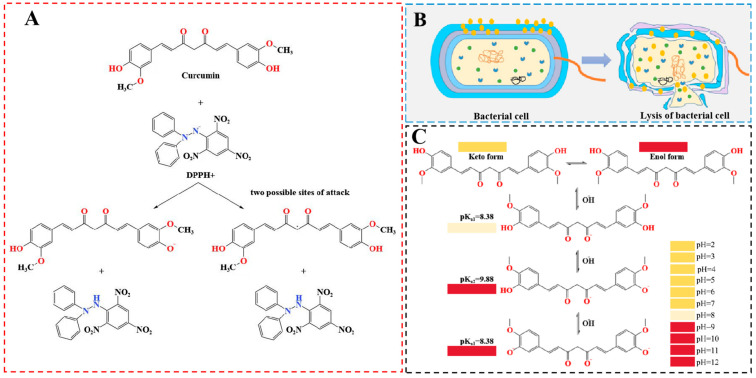
Chemical reaction between DPPH and curcumin (**A**) [63], antibacterial action of curcumin in film: schematic diagram of mechanism (**B**) [24], chemical structure changes of curcumin molecule in the different pH conditions (**C**) [67,68].

Curcumin is a naturally occurring ingredient and commonly used as an additive, displaying substantial antioxidant activity [60]. Comparative studies have demonstrated curcumin’s superior hydrogen peroxide scavenging capacity relative to conventional synthetic antioxidants (e.g., α-tocopherol, ascorbic acid, butylated hydroxyanisole (BHA), and butylated hydroxytoluene (BHT)) under equivalent conditions [63]. The reaction between curcumin and free radicals is shown in Figure 5A [48]. The antioxidant activity of curcumin is mainly due to the phenolic hydroxyl group, and a small fraction may be at the -CH_2_- position, which can bind with free radicals and provide a hydrogen atom [64]. Ma et al. (2017) prepared Tara gum films containing different contents of curcumin. With the increase in curcumin content, the DPPH free radical scavenging activity of the film was gradually enhanced, suggesting that the film had better antioxidant performance [61]. Liu et al. (2022) developed curcumin-encapsulated emulsion films that retained >44% DPPH scavenging capacity after 60-day storage, highlighting their long-term antioxidant stability [65]. Therefore, adding curcumin to the films was an effective way to improve the antioxidant performance [66]. Dos Santos Lima et al. (2025) prepared gelatin films with different modified curcumin (MC) concentrations by casting method. The modified curcumin obtained showed higher water solubility and antioxidant activity. Gelatin films containing 1% MC reduced the browning of banana peels during storage of bananas at 25 degrees Celsius and 75% relative humidity for 6 days [34].

### 3.2. Acting as an Antibacterial Agent

Food safety problems caused by foodborne pathogenic bacteria and outbreaks of various large-scale pathogenic infections have become a global public health problem. Foodborne pathogenic bacteria, such as *L. monocytogenes*, *E. coli*, and *S. aureus*, can cause vomiting, diarrhea, dizziness and, in severe cases, death [69]. The incidence rate and mortality of diseases caused by drug-resistant bacteria are increasing year by year [69,70,71]. As a result, it is important to explore food packaging with an antimicrobial function.

Curcumin has been demonstrated as an antibacterial agent. The antibacterial effect of curcumin is related to the function of temperature-sensitive filamentous mutant Z (FtsZ) required to inhibit bacterial cell division. Curcumin binds to the FtsZ protein, which blocks the assembly of FtsZ filaments, thus inhibiting cell division [72]. For example, curcumin inhibited the formation of *S. aureus* in vitro by inhibiting bacterial surface protein classifier A and intracellular fibronectin [73]. The possible mechanism of curcumin’s antibacterial activity is shown in Figure 5B [24]. In addition, the antibacterial effect of curcumin on Gram-negative bacteria is lower than that of Gram-positive bacteria, which is related to the structure and functional characteristics of the cell membrane [74]. The cell wall of Gram-positive bacteria contains phosphate groups, which can interact with curcumin, while the outer membrane of Gram-negative bacteria is rich in lipopolysaccharides, which can prevent the diffusion of curcumin [25,75]. In this regard, studies have shown that curcumin has a higher antimicrobial effect against Gram-positive bacteria than Gram-negative bacteria. Liu et al. (2021) studied the antibacterial properties of corn starch/polyvinyl alcohol-based films loaded with curcumin using bacteriostatic circle method. The results showed that the films could effectively inhibit the growth of *S. aureus*, *B. subtilis* and *E. coli* and, with the increase of curcumin content, the bacteriostatic circle became larger. In addition, the conclusion that the film has better antibacterial effect on Gram positive bacteria was also confirmed [76]. Li et al. (2024) prepared pectin/gelatin films loaded with curcumin and silver nanoparticles (AgNPs), and the inhibition rates against *Escherichia coli* and *S. aureus* were 99.57 ± 0.16% and 100%, respectively [77]. Choi et al. (2024) developed a smart indicator film consisting of microbial aliphatic polyester poly(3-hydroxybutyrate-4-hydroxybutyrate) (PHBC) and curcumin. PHBC was shown to have good UV protection, curcumin release, antioxidant and antimicrobial activities [29]. Therefore, curcumin demonstrates significant potential as a natural antibacterial component for advanced food packaging applications, particularly when combined with other antimicrobial agents.

### 3.3. Acting as a Monitor

Food deterioration will produce volatile gases, such as carbon dioxide (CO_2_), hydrogen sulfide (H_2_S), and ammonia (NH_3_), which will change the pH value of the environment inside the food package. The pH-sensitive indicators respond to these alterations through visible color transitions. Owing to its pH-responsive chromic properties, curcumin serves as an effective optical indicator for intelligent packaging systems [21,55,78,79,80]. The pH-indicating mechanism stems from curcumin’s β-diketone moiety undergoing keto–enol tautomerism, with the equilibrium between these forms being pH-dependent [75]. In acidic and neutral media, curcumin is mainly composed of the ketone type and its color is yellow. While under alkaline condition, curcumin is mainly composed of the enol type, where the color changes from yellow to red, and gradually deepens with the increase in pH value [81]. This chromic transition results from deprotonation of phenolic hydroxyl groups, generating phenolate anions that modify the electronic conjugation system (Figure 5C) [82]. Li et al. (2024) prepared pectin/gelatin film loaded with curcumin and AgNPs, which can display yellow (pH 3–8) to light red (pH 8–9) to dark red (pH 11–12) according to pH change, to monitor the freshness of shrimp packaging [77]. In addition, the color change of the films is related to the humidity in the environment. Ma et al. (2017) prepared an intelligent film by adding curcumin into the matrix of tala gum and polyvinyl alcohol (PVA). This acceleration occurs as adsorbed water mediates NH_3_ dissolution, forming NH^4+^ and OH^−^ that rapidly induce surface chromic transitions [61]. Mali and Pandey (2024) developed curcumin starch-based film pH-sensitive smart food packaging applications. These films show significant color changes when exposed to different pH environments, which can be effective for poultry freshness [55].

### 3.4. Acting as a Mechanical Property Enhancer

The incorporation of curcumin into biodegradable polymer matrices significantly enhances the mechanical performance of composite films, thereby expanding their potential for food packaging applications [83]. For instance, the addition of curcumin has been shown to increase the tensile strength and flexibility of konjac glucomannan (KGM)-based films [59]. These mechanical enhancements are critical for packaging integrity, ensuring resistance to mechanical stresses during processing, distribution, and storage while safeguarding contained food products [84]. Furthermore, the flexibility of the films is enhanced by curcumin, allowing them to bend and stretch without breaking. This is particularly beneficial for applications that require the material to conform to the shape of the food product, such as vacuum-sealed pouches or wraps for irregularly shaped items [24]. These mechanical improvements ensure that the packaging materials remain intact and functional, providing consistent protection for the food products from external physical impacts and contaminants. Therefore, curcumin-modified biodegradable polymer films offer dual advantages: superior mechanical performance and reliable food protection, making them promising candidates for diverse packaging applications ranging from fresh produce to processed foods [30,66,85]. Miao et al. (2024) selected cellulose with good biocompatibility and mechanical properties as a carrier and added high pH-responsive curcumin to develop a smart packaging material (RC/GC composite film) for real-time food safety monitoring. Compared with the pure cellulose film, the RC/GC composite film has excellent mechanical properties (4-fold improvement) and thermal stability (100 °C improvement) [14]. Mali and Pandey (2024) developed a curcumin starch based film for pH sensitive smart food packaging applications. These films show significant color changes when exposed to different pH environments, which can be effective for poultry freshness [55]. Zhang et al. (2025) prepared curcumin-containing cross-linked gelatin films using bis-formaldehyde carboxymethyl cellulose (OCMC) as a green cross-linking agent. A stable cross-linking network system consisting of imine covalent and hydrogen bonds was formed between OCMC and curcumin gelatin. As a result, the prepared cross-linked films exhibited excellent mechanical strength (breaking stress of about 18 MPa) and moisture resistance [54].

## 4. Curcumin-Loading Methods in Active-Intelligent Biodegradable Food Packaging

### 4.1. Loading Through Adding Directly

**Figure 6:**
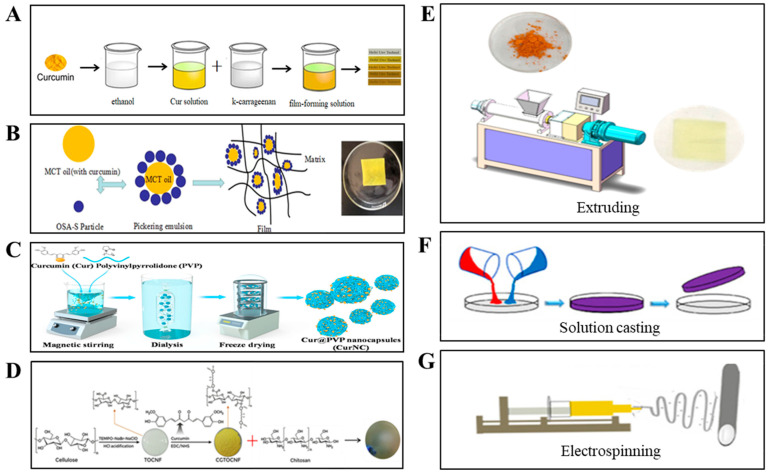
Curcumin-loading methods during the preparation of film: loading through adding directly (**A**) [67], loading through emulsification (**B**) [80], loading through curcumin nanoencapsulation (**C**) [55], loading through grafting (**D**) [89]. The preparation methods of extruding (**E**) [82], solution casting (**F**) [90], and electrospinning (**G**) [88].

Curcumin is slightly soluble in water and easily soluble in organic solvents (e.g., methanol, ethanol, acetone and isopropanol) and alkaline solutions [86,87,88], so curcumin can be dissolved in the above solution and directly mixed with the matrix to prepare films. This method is the simplest and fastest way to prepare curcumin loaded films, which have been widely used. Liu et al. (2018) dissolved curcumin in aqueous ethanol aqueous solution to prepare a k-carrageenan-based film (Figure 6A). The film had good barrier and mechanical properties, thermal stability, and was sensitive to pH value. It has been used to monitor the quality of fresh pork and shrimp [26]. Roy et al. (2020) dissolved curcumin in sodium dodecyl sulfate (SDS) and added it to a gelatin matrix to obtain the intelligent film. The results showed that curcumin can improve the UV-barrier properties of gelatin films and the antibacterial activity against *E. coli* and *L. monocytogenes*, as well as its antioxidant activity [73].

### 4.2. Loading Through Emulsification

Curcumin has low water solubility and instability, and cannot be uniformly dispersed in the hydrophilic material film matrix, significantly constraining its direct application in food packaging [91]. Emulsion-based materials produced from hydrocolloids and lipids result in better functionality than films produced with one component, especially with respect to their water barrier properties [20,92,93]. Many studies have shown that emulsion films can load bioactive compounds and improve the performance of bioactive films, especially for fat-soluble bioactive compounds. Consequently, curcumin-encapsulated emulsion systems can be integrated into film matrices to simultaneously enhance multiple functional properties while protecting curcumin from degradation [94,95]. Sanchez et al. (2022) prepared curcumin-loaded orange oil nano-emulsion by the emulsion phase inversion–emulsion phase inversion (EPI) method and then blended them with banana starch to form active packaging. Compared with banana starch film without adding curcumin-loaded nano-emulsions, the water vapor permeability decreased and the elongation at break increased [96]. Liu et al. (2021) mixed curcumin-loaded emulsions with corn starch and PVA to prepare pH sensitive films (Figure 6B). The results showed that, during the preparation and storage of the films, Pickering emulsion could effectively inhibit the decomposition of curcumin. Compared with the film loaded with curcumin through a direct approach, the curcumin-loaded emulsion film has stronger antibacterial properties and more pH-sensitive properties [76].

### 4.3. Loading Through Curcumin Nanoencapsulation

Recently, nanotechnology has emerged as a promising strategy among researchers, and nano-capsules can improve the chemical stability, hydrophilicity, sustained release and other properties of curcumin [97]. Xiao et al. (2021) fabricated curcumin/polyvinylpyrrolidone nano-capsules (CurNC) by the classical acid hydrolysis method and one-pot method (Figure 6C) and prepared soy protein isolate based films. The results showed that the release of curcumin was sustainable due to the more dense and homogeneous structure of the nanocomposite film [98]. Compared with the films prepared by adding curcumin directly, the antioxidant performance of the nanocomposite films was better, and the nanocomposite films showed more significant color response to pH buffer solution and NH_3_. In addition, the film has been applied to extend the shelf life of shrimp and monitor the freshness of shrimp in real time [98].

### 4.4. Loading Through Grafting

Although there have been many reports of improved curcumin and polymer composites, most are still based on physical mixing. Grafting small molecules onto the main or side chains of hydrophilic polymers is an innovative way to introduce molecules [99,100]. This can enhance the UV blocking, antioxidant, antibacterial and other properties of curcumin [89]. Zhang et al. (2021) prepared a composite film of chitosan and curcumin grafted tempo TEMPO-oxidized cellulose nanofibers (CGTOCNF) through esterification, which can improve the stability of curcumin (Figure 6D). The results showed that the addition of CGTOCNF improved the UV barrier performance, water solubility, antioxidant and antibacterial properties of the composite film as compared with pure chitosan film. Therefore, the grafting approach is an effective way to load the bioactive compounds onto the films [89].

### 4.5. Loading Through Cyclodextrin

Curcumin shows biological activities, such as antioxidant and anti-inflammatory activities, but its properties, such as very poor water solubility and easy decomposition in the presence of light, limit its application in the food and pharmaceutical fields [46,49]. Cyclodextrin (CD) is a water-soluble cyclic oligosaccharide compound consisting of a series of 6 (α-cyclodextrin), 7 (β-cyclodextrin) or 8 (γ-cyclodextrin) D-glucose monomers linked by α-1-4 glycosidic linkages with a hydrophobic cavity structure [101], which has been a hotspot in drug delivery research due to the advantages of a simple preparation process and easy accessibility materials [102]. Curcumin molecules are less polar and more hydrophobic, while β-CD has an external hydrophilic and internal hydrophobic cavity structure, therefore the use of β-CD as a negative carrier for curcumin has the advantage of its chemical structure, and curcumin molecules are able to spontaneously enter into the cavity of β-CD and form a delivery system by self-assembly [103].

Arya and Raghav et al. (2021) [102] utilized β-cyclodextrin and curcumin to form an inclusion complex, which increased its aqueous solubility by 206-fold. This study also found that the curcumin in the inclusion complex could be continuously released in vitro for 5 h. Unreleased curcumin could be protected in the β-CD cavity before the released curcumin could bind to the enzyme or receptor. Hedi et al. (2021) [104] utilized epichlorohydrin as a cross-linking agent and grafted γ-cyclodextrin onto bovine serum protein (BSA) to obtain γ-cyclodextrin-BSA nanoparticles and loaded with curcumin. The results showed that the pH stability and salt stability of γ-cyclodextrin-BSA were improved compared with that of γ-cyclodextrin. The in vitro release study found that curcumin was released slowly in pH 1.2 hydrochloric acid and faster in pH 7.2 neutral environment, indicating that γ-cyclodextrin-BSA nanoparticles were able to protect curcumin from being released in the gastric environment and in the intestinal tract. Not only can this effectively improve the water solubility of curcumin, but also protect it from being damaged by the gastric acid environment.

However, due to the poor water solubility of β-cyclodextrin itself, the simple use of β-cyclodextrin as a carrier for curcumin is not satisfactory in terms of effectiveness. Therefore, the introduction of different functional groups, such as methyl, hydroxypropyl, carboxymethyl, and sulphobutyl into cyclodextrins can lead to the formation of cyclodextrin derivatives with higher stability, better water solubility, and better molecular recognition ability (cyclodextrin and its derivatives (CDs), which modify the surface of cyclodextrins and improve their solubility in water [103]. For example, cyclodextrin succinate (SACD) was modified by dry-heat esterification to increase curcumin loading from 15.7% to 91.7% and significantly improve its photo-thermal stability [103]. Liu et al. (2022) [105] modified the structure of curcumin with boron trifluoride ether, and then embedded it into the hydrophobic cavities of α-cyclodextrin and hydroxypropyl β-cyclodextrin, respectively, to form inclusion complexes, and the results showed that both inclusion complexes could exhibit the activity of inhibition against the proliferation of cancer cells.

## 5. Preparation Methods of Active-Intelligent Biodegradable Food Packaging with Curcumin

### 5.1. Extrusion

The extrusion method of film formation is to mix curcumin with the matrix without any treatment. The specific operations are as follows: (a) Select the appropriate temperature, pressure and screw speed; (b) Mix all the components of the film in the extruder, and then extrude the produced material into a film with a hot press [57,106]. Although this method is easy to operate and can be applied to the field of commercial food packaging, it is only suitable for thermoplastic polymers, such as starch and polylactic acid (PLA) [107,108]. Reddy et al. (2019) mixed curcumin powder with poly lactic acid particles and then prepared the film through a twin-screw extruder and hot pressing mechanism. They found that the films added with curcumin showed greater barrier properties and increased surface hydrophobicity and UV resistance [109]. Zhai et al. (2020) also prepared curcumin/low density polyethylene hydrophobic film by an extrusion method (Figure 6E). The film had good stability in buffer solutions with different pH values and was sensitive to ammonia [82].

### 5.2. Solution Casting

The solution casting method has advantages of low cost, simple operation and fast speed, and is applied to the preparation of food packaging film at laboratory scale. The main operation is to add the solution containing the pH sensitive indicator into the polymer solution prepared in advance, stir it evenly, and then pour the degassed film-forming solution into the Petri dish or other flat surface, and control the temperature and humidity of the film drying (Figure 6F) [57,63]. Xie et al. (2020) prepared active films consisting of potato whole peel combined with bacterial cellulose and curcumin using the solution casting method. The results showed that the addition of curcumin further improved the antioxidant properties of the composite films. The composite films with curcumin addition also significantly reduced lipid oxidation and the malondialdehyde (MDA) content of pork [60]. Subbuvel et al. (2022) prepared polylactic acid (PLA) film containing curcumin and fenugreek essential oil through a solution casting method; the performance of the composite film was improved and used to monitor the quality of strawberry slices [110].

### 5.3. Electrospinning Technology

Electrospinning technology is a special fiber manufacturing process with simple operation and mild conditions [22,88]. Charged fluid mainly flows and deforms through electrostatic field to form fibrous materials. Fiber materials are formed by electrostatic field flow and deformation, and the obtained nanofibers have good flexibility, porosity and high specific surface area, which are beneficial to the encapsulation of bioactive compounds [22,88]. At present, electrostatic spinning technology has been successfully applied to intelligent packaging. Yildiz et al. (2021) prepared an electro-spun nanofiber salt pH sensor film using chitosan/polyethylene oxide as the external support matrix of curcumin to monitor the freshness of chicken (Figure 6G) [81]. Luo et al. (2020) dispersed curcumin in film-forming solution to form an electrospinning solution. Curcumin-loaded film was prepared by free surface electrospinning method, which was used for the detection of amine compounds [111].

## 6. Application of Curcumin-Loaded Active-Intelligent Food Packaging

As consumers aware of the health and environmental problems that may be brought about by the use of plastic food packaging, there is growing research on films that combine multi-functionality, such as anti-oxidation, anti-bacterial and intelligent responsiveness [112,113]. Curcumin has been added to explore the innovative food packaging due to its excellent properties, such as its anti-inflammatory, antioxidant, antibacterial and pH-sensitive properties [21,29,55,114,115,116,117]. In order to show the potential of active-intelligent packaging materials in the development of food industry, we will focus on its application in food packaging in this section. Table 1 shows some examples of the key properties and applications of curcumin-loaded films. The main applications of curcumin-loading films in the food industry have been summarized as follows.

### 6.1. Application for Meat Packaging

Meats like pork, beef and chicken are rich in protein, but protein-rich food is prone to chemical and microbial corruption during storage, which reduces the nutritional value of the food [116,117,126,127,128]. An important reason for meat deterioration is the volatile components produced by microorganisms, such as ammonia, methylamine, dimethylamine, trimethylamine and other similar compounds, known as total volatile basic nitrogen (TVBN) [82,129]. TVBN is commonly used to evaluate the quality of meat, and active-intelligent food packaging containing curcumin has been applied to meat.

Xie et al. (2021) loaded different contents of curcumin onto pectin/chitosan film to prepare pH-sensitive packaging. As for the change of malondialdehyde (MDA), a marker of lipid peroxidation in pork, compared with film without curcumin, the MDA of pork with curcumin was lower, indicating that the film containing curcumin had good antioxidant performance. In addition, due to the addition of curcumin, the UV barrier performance of the film is improved, so this reduces the impact of light on the quality of pork. The film containing curcumin did not significantly change the color, taste and pH of pork, which confirmed that curcumin composite films can protect pork and extend its shelf life [64]. Liu et al. (2022) prepared chitosan/gelatin based films with Pickering emulsion and curcumin as additives, and the composite films were applied to pork. After 3 days of pork storage, the color of the film gradually changed from bright brown to saddle brown. According to the National Standard of Food Safety of China(GB 2707-2016) [130], when the TVBN of meat exceeds 15 mg/100 g, this means that it was rotten. Despite this, the TVBN (30.52 mg/100 g) coated with the composite film was lower than that of the control group, indicating that the composite film has a protective and monitoring effect on meat [65]. Similarly, Zhai et al. (2021) prepared curcumin/low density polyethylene (LDPE) films by extrusion, which can also monitor the freshness of beef effectively [82]. Huda et al. (2025) developed a packaging film containing ethanol extract of turmeric, which showed excellent antibacterial activity and significantly reduced the total number of colonies in chicken minced meat [131]. The konjac glucomannan/carrageenan/curcumin/anthocyanin film prepared by Zhou et al. (2021) was used to monitor the freshness of chicken during storage [132]. Sun et al. (2024) designed smart pH- and ammonia-sensing edible films based on isolated whey protein–cellulose nanocrystalline biopolymers by incorporating different functional colorants (curcumin, phycocyanin, and modified lycopene) for pork freshness monitoring analysis. The composite colorant film undergoes a noticeable color change when the meat spoils. In addition, meat packaged with the combination colorant film exhibited lower levels of lipid oxidation compared to meat packaged with the single colorant film [116].

### 6.2. Application for Seafood Packaging

Seafood, such as fish, shrimp and crab, is an important source of long-chain polyunsaturated fatty acids, vitamins and minerals, which are beneficial to human health [133], while these foods are also perishable. Under the action of microorganisms, high levels of TVBN will be produced, and the pH value will also change. Therefore, packaging materials containing pH-sensitive infectious materials can be used to monitor the quality changes of seafood during storage [50,117,134].

Vadivel et al. (2019) monitored the freshness of fish according to the existence of TVBN in packaging and prepared a biodegradable food packaging composite film material with curcumin, as an indicator to evaluate the freshness of fish. After the fish was stored for 10 h, the color of the film changed to orange–red, and the TVBN reached 54.5 mg/100 g. It can be seen from China’s National Food Safety Standard [135] (GB2733-2015) that the TVBN of the fish exceeded 20 mg/100 g, which indicates that it was rotten. Furthermore, the high antioxidant activity of the film could inhibit the oxidation of the product to a certain extent [136]. Ezati et al. (2020) mixed curcumin and sulfur nanoparticles with pectin to prepare pH-responsive films. The film was used to monitor the quality of shrimp. During the storage of shrimp, the pH value of shrimp increased from 6.3 to 7.1, and the film changed from yellow to orange. Moreover, the composite film had high UV performance, high water contact angle, good thermal stability and strong antioxidant activity. The film also had inhibitory effect on *E. coli* and *L. monocytogenes* [79]. Taghinia et al. (2021) prepared films by adding curcumin to *Lallemantia iberica* seed gum to evaluate the freshness indication effect of the composite film on shrimp. After 5 days of shrimp storage, the TVBN of shrimp reached 43.89 mg/100 g, the a* value (red degrees) of the film increased from 5.7 to 9.9, and the color changed from yellow to red. In addition, the composite film had excellent antibacterial/mold-resistant and antioxidant properties [50].

### 6.3. Application for Fruit and Vegetable Packaging

Fresh fruits and vegetables experience affect to their shelf life due to loss of moisture, microbial growth, and other factors [137]. To extend the shelf life of fruits and vegetables, various methods have been developed, such as refrigeration, modified atmosphere packaging (MAP) with increased CO_2_ concentration, and paraffin-based reactive coatings [138,139]. However, these methods are expensive, time-consuming and affect the flavor and appearance of fruits and vegetables [140]. Therefore, it is important to explore cost-effective and green methods to extend the shelf life in order to reduce the waste of fruits and vegetables.

Liang et al. (2022) prepared a biological multifunctional film loaded with curcumin. The composite film had excellent antioxidant and antibacterial properties and can effectively prevent fruit rot. Compared with the control group, the shelf life of pitaya and avocado was extended by at least 4 days [141]. Joshy et al. (2020) prepared lipid polymer hybrid nanoparticle dispersions loaded with curcumin as a protective coating for fresh fruits and vegetables. After tomatoes and apples were stored for 12 days, the weight loss of coated tomatoes was 6.8% and the weight loss of coated apples was 3.4%, both lower than that of uncoated samples. It was demonstrated that the coating can protect the quality of fruits and vegetables [142]. Curcumin-loaded film was also used to protect the freshness of pineapple, strawberry, banana and kiwifruit [18,93,143]. Dos Santos Lima et al. (2025) prepared modified curcumin gelatin film, and the results showed that 1% MC gelatin film can effectively reduce banana peel browning during storage [34]. In addition, packaging systems loaded with curcumin are used to preserve fruit juice. Wu et al. (2018) developed a gelatin/β-cyclodextrin/curcumin intelligent film. The results showed that β-cyclodextrin/curcumin could improve the antioxidant activity of gelatin film, and the composite film could effectively prevent the browning of apple juice, which could be used for its preservation [144]. Surprisingly, for the film containing 5 mg curcumin, compared to the film containing 2.5 mg curcumin, at higher concentrations, the structure of the film becomes loose, weakening the barrier of the film to oxygen. The composite film had great potential for the packaging of apple juice [144]. Luo et al. (2025) prepared a light-responsive thermally controlled curcumin-releasing packaging film by combining chitosan, Cu-Mox and curcumin (CS/CMC). Oranges treated with CS/CMC film had a longer shelf life and less nutrient loss than polyethylene (PE) film, indicating that CS/CMC0.02 has good potential as a packaging film [56].

### 6.4. Application for Oil Packaging

During storage, oils are easily affected by air, temperature, oxygen, light, metal ions and their own fatty acid components, which cause complex chemical changes and lead to deterioration. In order to prevent food spoilage and protect human health, it is very important to control oil oxidation in food [145,146,147]. Campos et al. (2019) explored the protective effect of extruded film containing curcumin on oil. The accelerated oxidation test (60 °C, 7 days) showed that the tocopherol concentration of chia oil protected by the film was high, i.e., the film containing curcumin can effectively prevent oil degradation [123]. Zhang et al. (2022) introduced curcumin into castor oil-based waterborne polyurethane and compounded it with gelatin to form a composite film. This was used to protect soybean oil. After 50 h UV irradiation, the color change of soybean oil covered with composite film was not obvious, and peroxide value and thio-barbituric acid were significantly lower than in control group. Therefore, the film has potential in photooxidation-resistance packaging [100]. Demircan et al. (2025) prepared a bilayer film of PVA and sodium alginate (SA) loaded with curcumin (Cur–PVA/SA). The results showed that the oil packaged in Cur–PVA/SA pouches would remain stable for 12 months at 23 °C [147].

## 7. Impact of Curcumin Migration from Active Packaging on Food Safety

Functional packaging systems extend food preservation periods while improving product integrity and safety profiles via regulated emission of bioactive compounds exhibiting antimicrobial or antioxidant properties. Scientific investigations have established that the transfer of substances from packaging substrates into edible commodities and their simulated counterparts operates through well-characterized physicochemical mechanisms [148]. According to Fick’s equations of diffusion, mass transfer from plastic materials to food generally occurs [148,149]. Studies investigating the impact of cellulose nanocrystals (CNCs) on curcumin migration from bio-composite films showed that a less significant release was observed during 12 to 36 h, which may be due to the interaction of CNC with curcumin through adsorption onto the surface, reducing free hydroxyl groups and providing better compatibility within the poly(lactic acid) matrix [150]. Curcumin incorporated into PVA/SA films contributed to the delayed lipid oxidation and its release followed a biphasic diffusion model to ensure prolonged antioxidant activity [147]. Through covalent functionalization via thermomechanical processing, curcumin was chemically immobilized onto polypropylene substrates to engineer migration-resistant smart packaging systems. Validation through standardized migration testing protocols demonstrated a maximum release of 0.011 mg/cm^2^, registering at 89% below the European Commission’s regulatory threshold (0.1 mg/cm^2^) for food-contact substance migration [151].

## 8. The Possibility of Curcumin Release into Packaged Food Products, the Stability of Packaging over Time, and Its Biodegradability

### 8.1. Curcumin Release into Packaged Food Products

The release of curcumin from packaging materials into food products is influenced by factors such as the type of food matrix, pH conditions, and the presence of surfactants or polymers. Studies have shown that curcumin release can be enhanced under alkaline conditions due to the solubility of certain polymers, such as carboxymethyl cellulose, which swell in alkaline media and facilitate curcumin release into the gastrointestinal tract [152]. Additionally, the use of nano-emulsions and microgels has been demonstrated to prolong curcumin release, potentially enhancing its bioavailability and functional effects [153].

### 8.2. Stability of Packaging over Time

The stability of curcumin-enriched packaging materials is crucial for maintaining their functional properties during storage. Research indicates that lipid-based nano delivery systems, such as nanostructured lipid carriers (NLCs) and solid lipid nanoparticles (SLNs), exhibit higher stability when subjected to different food simulants. However, the long-term stability of these systems may vary depending on the specific formulation and environmental conditions. For instance, curcumin-loaded films made from poly (lactic acid) and sodium carboxymethyl cellulose have shown improved mechanical and UV-barrier properties, but their water vapor permeability and tensile strength may be negatively affected over time [4].

### 8.3. Biodegradability of Packaging Materials

The biodegradability of curcumin-enriched packaging is an important consideration for environmental sustainability. Studies on biopolymer films, such as those based on chitosan and cellulose nanofibers, have demonstrated their effectiveness in reducing microbial counts and lipid oxidation in stored food products [154]. These materials are also environmentally friendly, as they can be fully degraded under appropriate conditions. However, further research is needed to optimize their mechanical properties and ensure their practical applicability in commercial food packaging. While curcumin-enriched packaging offers promising benefits for food preservation and functional food development, a more detailed exploration of curcumin release mechanisms, packaging stability over time, and biodegradability is essential to fully understand its potential applications and limitations [155]. Future research should focus on addressing these gaps to enhance the practical utility of curcumin-enriched packaging in the food industry.

## 9. Future Work and Challenges

Curcumin-incorporated films represent a promising intelligent packaging system that enables nondestructive quality monitoring while simultaneously preserving food freshness and enhancing safety parameters. This system has been used in seafood, meat, fruits, etc. Despite curcumin’s multifunctional advantages, several technical challenges must be addressed to facilitate commercial-scale adoption. First, curcumin exhibits inherent instability and susceptibility to degradation. To enhance its performance, strategies such as combining it with antimicrobial nanoparticles, essential oils, metal ions, or encapsulating it within emulsion systems have been explored to improve stability [19,116,156,157]. However, prior to the commercial adoption of curcumin-based packaging systems, comprehensive food toxicological studies are imperative to further validate safety profiles [158].

Additionally, the limited colorimetric response range of curcumin necessitates further research to optimize the pH sensitivity of curcumin-loaded films [14]. For instance, integrating curcumin with other pigments, such as anthocyanins, could broaden the color change spectrum in smart films, thereby enabling more precise monitoring of food freshness. Current research remains largely confined to laboratory-scale investigations, with limited manufacturing technologies hindering large-scale packaging commercialization [159]. Consequently, future efforts should focus on advancing film fabrication techniques and developing intelligent applications. For example, consumers could utilize smartphones or other devices to accurately interpret color changes in films, accessing real-time information on food quality and status [4,5,11,58,63]. Furthermore, enhancing the safety and sustainability of food packaging technologies is critical. Finally, the high production costs of these packaging materials significantly impede their commercial viability [6]. Consequently, future efforts should focus on advancing film fabrication techniques and developing intelligent applications.

Furthermore, enhancing the safety and sustainability of food packaging technologies is critical. Finally, the high production costs of these packaging materials significantly impede their commercial viability [108]. Thus, additional studies are required to identify cost-effective materials and optimize processing methods to reduce both material and manufacturing expenses.

## Figures and Tables

**Table 1:** Antioxidant and antibacterial properties and applications of active-intelligent food packaging.

Matrix	Form of Curcumin Incorporation to the Polymer Matrix	Antioxidant	Antibacterial	Application	Ref.
Chitosan	Casting method using ethanol solution	-	The curcumin-added film significantly reduced the number of *S. aureus* and *Salmonella* during a 3-h exposure period	Promising application for food packaging	[118]
Gelatin	Casting method using SDS solution	The scavenging activity of DPPH and 2,2′-azino-bis(3-ethylbenzothiazoline-6-sulfonic acid) (ABTS) increased to 76.2% and 88.1%, respectively, when gelatin was mixed with 1.5 wt% curcumin, showing very effective antioxidant activity	Gelatin/curcumin composite film showed certain antibacterial activity against food-borne pathogens *E. coli* and *L. monocytogenes*	Ensure food safety and extend food shelf life	[66]
Gelatin	Solution casting method using ethanolwater mixture	Adding curcumin can significantly improve the oxidation resistance of the film	Gelatin-curcumin films showed no antimicrobial activity against *Salmonella enteritidis*, *E. coli*, *Bacillus cereus* and *Staphylococcus aureus*, a result that can be attributed at least in part to the low curcumin concentrations used in the films studied	Intelligent food packaging and reflect the quality of product based on the capacity to sense pH change	[119]
Pectin/chitosan	Solution casting method using water (containing 1 mL deep eutectic solvent (DES))	The DPPH and ABTS radical inhibition rates of the composite film were 58.66% and 29.07%, respectively	The film has excellent bactericidal performance and inhibits the growth of microorganisms	Monitor the quality of pork	[64]
Polylactic acid (PLA)/fenugreek essential oil	Solution casting method	The ABTS and DPPH scavenging activity of curcumin-loaded films increased significantly to 63.8% and 53.5%, respectively	PLA/Cur film had certain antibacterial activity against foodborne pathogenic bacteria, *E. coli* and *S. aureus*	Extend shelf life of strawberries	[110]
Carboxymethyl cellulose	Solution casting method using distilled water	The composite film added with curcumin showed strong antioxidant activity. When 1 wt% curcumin was added, its scavenging activity was significantly increased to 40.2% and 92.5%. The increase in antioxidant activity of the curcumin composite film depends on the concentration of curcumin	The composite film added with curcumin showed antibacterial effect on *E. coli* and *L. monocytogenes*, and the growth of the tested bacteria was delayed	Prevent food photooxidation, ensure food safety and prolong food shelf	[73]
Tara gum/polyvinyl alcohol (PVA)	Solution casting method using NaOH solution	The DPPH scavenging capacity was increased from 1.81% to 35.16% as the curcumin content increased to 5%	-	Yield new designs for the protection of fatty foods.	[61]
Starch extracted from proso millet	Solution casting method using NaOH solution	The DPPH and ABTS of proso millet (PMS), with curcumin content of 3%, were 100% and 42.96%, respectively		New active packaging materials	[120]
Whey protein isolate (WPI)	Solution casting method	The films enriched with curcumin presented good antioxidant properties	-	Fabricate bioactive edible antioxidant films	[121]
Pistachio green pectin/poly vinyl alcohol (PVA)	Solution casting method		The PGHP/PVA/curcumin films showed a clear antibacterial activity against *E. coli* and *S. aurous* (with inhibition zones of 6.3 mm and 4.7 mm, respectively)	Monitor the quality of fish	[122]
Thermoplastic cassava starch (TPCS)/poly(butylene adipate-co-terephthalate) (PBAT)	Solution casting method using blow extrusion	The film containing curcumin had antioxidant activity	The addition of curcumin enhances the antibacterial activity against Gram-positive (*S. aureus*) and Gram-negative (*P. aeruginosa* and *E. coli*) bacteria	Application in normal sub oil packaging was verified	[123]
Carboxymethyl cellulose/gelatin/curcumin/chitosan	Solution casting method using emulsion	-	It has inhibitory effect on *E. coli* and *S. aureus*	Monitoring the qality of pork during storage	[92]
Gallic acid/quercetin	Nano emulsions (NE)	Curcumin NE-loaded gelatin composite films exhibited the highest antioxidant activity (27.20, 45.9 and 60.51%) at 5%, 10% and 20% NE concentrations, respectively	Curcumin-gelatin film had antibacterial activity against *S. typhimurium* (6.97mm) and *E. coli* (7.47 mm)	Extend shelf life of fresh broilers to 17 days	[124]
Zein	Electrostatic spinning		The composite film had antibacterial activities against *E. coli* and *S. aureus*, and the inhibition efficiency increased with the increase of curcumin content	Potential for antibacterial applications	[91]
Konjac glucomannan and zein	80% ethanol solution for electrospinning	The DPPH scavenging activity of the film increased by about 15%	The film containing curcumin has good bacteriostatic effect on *E. coli* and *S. aureus*, and the bacteriostatic range is about 12–20 mm	Potential application in food packaging	[125]
